# Overexpression of IL-10 in C2D Macrophages Promotes a Macrophage Phenotypic Switch in Adipose Tissue Environments

**DOI:** 10.1371/journal.pone.0086541

**Published:** 2014-01-21

**Authors:** Linglin Xie, Qiang Fu, Teresa M. Ortega, Lun Zhou, Dane Rasmussen, Jacy O’Keefe, Ke K. Zhang, Stephen K. Chapes

**Affiliations:** 1 Department of Basic Sciences, School of Medicine and Health Science, University of North Dakota, Grand Forks, North Dakota, United States of America; 2 Division of Biology, Kansas State University, Manhattan, Kansas, United States of America; 3 Departments of Gerontology and Oncology, Tongji Hospital, Huazhong University of Science and Technology, Wuhan, China; 4 Department of Pathology, School of Medicine and Health Sciences, University of North Dakota, Grand Forks, North Dakota, United States of America; 5 North Dakota IDeA Network of Biomedical Research Excellence Bioinformatics Core, University of North Dakota, Grand Forks, North Dakota, United States of America; The First Affiliated Hospital of Nanjing Medical University, China

## Abstract

Adipose tissue macrophages are a heterogeneous collection of classically activated (M1) and alternatively activated (M2) macrophages. Interleukin 10 (IL-10) is an anti-inflammatory cytokine, secreted by a variety of cell types including M2 macrophages. We generated a macrophage cell line stably overexpressing IL-10 (C2D-IL10) and analyzed the C2D-IL10 cells for several macrophage markers after exposure to adipocytes compared to C2D cells transfected with an empty vector (C2D-vector). C2D-IL10 macrophage cells expressed more CD206 when co-cultured with adipocytes than C2D-vector cells; while the co-cultured cell mixture also expressed higher levels of *Il4, Il10*, *Il1β* and *Tnf*. Since regular C2D cells traffic to adipose tissue after adoptive transfer, we explored the impact of constitutive IL-10 expression on C2D-IL10 macrophages in adipose tissue *in vivo*. Adipose tissue-isolated C2D-IL10 cells increased the percentage of CD206^+^, CD301^+^, CD11c^−^CD206^+^ (M2) and CD11c^+^CD206^+^ (M1b) on their cell surface, compared to isolated C2D-vector cells. These data suggest that the expression of IL-10 remains stable, alters the C2D-IL10 macrophage cell surface phenotype and may play a role in regulating macrophage interactions with the adipose tissue.

## Introduction

Macrophages are a heterogeneous group of cells which have different functions, morphologies and phenotypic properties depending on the microenvironment in which they reside [1], [2], [3], [4], [5]. The microenvironment is regulated by the purpose of the tissue, genetic background of the animal and the immune status of the host [1]. For example, adipose tissue macrophages (ATMs) consist of at least two different phenotypes: classically activated M1 macrophages and alternatively activated M2 macrophages [6], [7], [8], [9], [10]. M1 or M2 ATMs are identified by the presence or the absence of CD11c [8], [11], [12]. ATMs have been characterized as an anti-inflammatory (M2)-dominant phenotype in lean mice, while there is an increased number of M1-phenotype macrophages in obese subjects [6], [8], [9], [10], [13]. It is still unclear if the phenotypic switch of ATMs between M1 and M2 is a consequence of the host having more adipose tissue or if the increased number of M1 cells is an active cause of environmental alteration such as an increased amount of Leptin [14]. Therefore, important questions remain about ATM signaling leading to changes in macrophage phenotypes and how this change impacts the adipose tissue microenvironment.

Interleukin 10 (IL-10) is a Th_2_-type (M2) anti-inflammatory cytokine [15]. The principal function of IL-10 is to limit and ultimately terminate inflammatory responses [15]. The anti-inflammatory effects of IL-10 involves both inhibition of pro-inflammatory cytokine synthesis (e.g. IL6, IL1β and TNF-α) and repression of bioactivity in target cells [15], [16]. In adipose tissue, IL-10 signals by binding of the IL-10 receptor and activation of JAK/STAT pathway to regulate cytokine secretion [17], [18], [19]. In isolated adipocytes and skeletal muscle, IL-10 enhances insulin sensitivity by increasing glucose uptake and antagonizing TNF-α [7], [20]. However, *in vivo* physiological data about IL-10 are inconsistent. Transgenic mice with overexpressed IL-10 in muscle (MCK-IL-10) prevented high fat diet-induced inflammation as measured by decreased recruitment of skeletal muscle macrophages and lower levels of skeletal muscle IL-6, TNF-α and IL-1β [20]. In contrast, systemic overexpression of IL-10 using an adenovirus vector increased expression of M2 macrophage markers in epididymal fat tissue of both lean and obese mice, but did not affect the level of *Mcp1, Tnf,* and *Il6* [10]. Thus, the role of IL-10 on macrophage phenotype and activation remains unclear.

We previously reported that C2D macrophage cells reside early in the macrophage lineage *in vitro* [21], but acquire a different phenotype depending on the type of adipose tissue that they traffic to, after adoptive transfer into the peritoneal cavity [4], [5]. Because the adoptive transfer of C2D macrophages can ameliorate the impact of infection [22] and C2D macrophages trafficked to adipose tissue [4], we wondered if the cells would be able to influence the adipose tissue environment if they overexpressed IL-10. Therefore, we constructed a C2D macrophage cell line that stably overexpresses IL-10 (C2D-IL10). In the present study, we investigated the impact of constitutive expression of IL-10 on C2D macrophages and how the constitutive expression of IL10 impacts the C2D macrophage phenotype when those macrophages interact with adipocytes *in vitro* and *in vivo*.

## Materials and Methods

### Mouse Strains

C57BL/6J (B6) mice were obtained from the Jackson Laboratory (Bar Harbor, ME). Male and female, 8–16 week-old mice were bred in the rodent facility of the University of North Dakota and used in these experiments. Mice were fed a normal mouse chow diet and were allowed to feed Ad libitum. Mouse experiments were completed according to a protocol reviewed and approved by the Institutional Animal Care and Use Committee of the University of North Dakota, in compliance with the USA Public Health Service Policy on Humane Care and Use of Laboratory Animals.

### Antibodies

The anti-Mouse F4/80 antigen (PE-Cy7 labeled) and anti-mouse CD11c (PE labeled) were purchased from eBioscience (San Diego, CA). A rat anti-mouse CD206 (Alexa Fluor® 647 labeled), a rat anti-mouse CD301 (Alexa Fluor® 647 labeled), A rat anti-mouse Ly6B.2 Alloantigen (Alexa Fluor® 700 labeled) and a rat anti-mouse CD11b (Alexa Fluor® 647 labeled) were from AbD Serotec (Raleigh, NC). Recombinant mouse IL-10 was from eBioscience (San Diego, CA).

### Construction of C2D-IL10 Cells

The full length mouse *IL-10* coding sequence was amplified from LPS-treated (10 µg/ml) peritoneal cavity macrophages and was flanked with *ApaI* restriction sites on both 5′ and 3′ ends, using the following primer sets: *5′-AA GTT GGGCCC ATG CCT GGC TCA GCA CT-3′* (forward) and *5′- C GCG GGGCCC GTA GCT TTT CAT TTT GAT CAT CAT GTA TGC-3′* (reverse). The cDNA was inserted into the pCR4-TOPO vector and the insert was sequenced to ensure homology to the mouse IL-10 sequence from NCBI database (NM_010548.2). The insert was then cloned into the pAcGFP1-N1 mammalian expression vector (kind gift from Dr. Sandy Beeser, Kansas State University). The purified IL-10 plasmid (pAcGFP1-N1) was used to transfect C2D macrophages using the lipofectamine reagent according to the manufacturer’s instructions (Invitrogen). IL-10 overexpressing C2D macrophage cells were selected by growth in G418 (400 µg/ml) and constitutive expression of the IL-10 transcript was verified by RT-PCR. The level of biologically active IL-10 was determined by ELISA. C2D-vector alone cells were also constructed in parallel to act as controls in these studies.

### Cell Culture

The C2D cell line was created by our group and was cultured in DMEM_4_ as described previously [21], [23], [24].

3T3-L1 adipocytes were obtained from the American Type Culture Collection (Manassas, VA). Adipocytes were cultured and differentiated as described previously [25], [26].

Direct co-culture of 3T3L1 adipocytes and C2D-IL10 or C2D-vector cells were performed by directly adding C2D-IL10 or C2D-vector (0.5×10^5^ viable cells; trypan blue exclusion) to 12-well plates in DMEM_10_ containing 4 X 10^5^ 3T3L1 cells that had been differentiated for 8 days. Macrophages were incubated with 3T3L1 cells for four days and did not appear apoptotic or necrotic after the 4-day incubation period, as assessed by light microscopic examination.

### Adoptive Transfer of CFDA-SE Labeled Cells

C2D-IL10 or C2D-vector cells were suspended in sterile, prewarmed (37°C) PBS at a concentration of 1.5×10^6^ per ml. Cells were stained with carboxyfluorescein diacetate succinimidyl ester (CFDA-SE) according to the manufacturer’s protocol and as described before [4].

### Stromal Vascular Cell (SVC) Isolation and FACS Analysis

Epididymal fat pads were minced and incubated in pre-warmed (37°C) DMEM containing 1mg/ml collagenase and 5 mM CaCl_2_. Thereafter, the samples were incubated for 45 min at 37°C with constant shaking at 60 rpm. The adipose tissue cells were passed through a 100 µm cell strainer. Cells were then centrifuged at 370×g for 1 minute to separate the adipose tissue and the SVC containing injected C2D-IL10 or C2D-vector cells. Cells were fixed in 2% paraformaldehyde in PBS for 20 min at 37°C and we stained the cells for flow cytometry analysis as we have described previously [5].

### Cell Sorting and Flow Cytometry Analysis

Fresh isolated SVC containing injected C2D-IL10 or C2D-vector cells were re-suspended in PBS. Cell sorting was based on C2D macrophage cell CFDA-SE fluorescence, with the lowest 10% of the positive cells not selected. Cell sorting was performed with a BD FACSAria III flow cytometer. Cells were sorted at a rate of 15,000 cells per second and approximately 1×10^5^ viable (trypan blue exclusion), positive cells per group were collected on ice and centrifuged at 350×g for 5 min at 4°C for RT-PCR analysis.

For the analysis of the surface molecules, cells were incubated with the specific antibody or isotype control diluted in Hanks buffered salt solution (HBSS) for 30 min in the dark at 4°C. After two washes with HBSS, cells were fixed in 1% formalin. Labeled cell surface proteins were assessed by BD LSR II flow cytometer.

### Cytokine Detection

Overnight culture medium from the 3T3L1 adipocytes, C2D-IL10, C2D-vector cells or co-cultures was collected for the cytokine detection of IL6, IL-10, IL1β and TNF-α. The cytokines were measured by ELISA according to the manufacturer’s protocol (eBioscience, San Diego, CA). Sensitivity of the assays for TNF-α, IL-6, IL-1β, IL-10, were 8 pg/ml, 4 pg/ml, 8pg/ml and 30pg/ml, respectively. Standard curve-based concentrations lower than the sensitivity range was defined as “undetectable”. The assays were quantified spectrophotometrically using a Bio-Rad microplate reader, model 680 (Bio-Rad Laboratories, Inc., Hercules, CA) at 450 nm.

### Realtime-PCR Analysis

Total RNA was extracted using Trizol reagent (Invitrogen). cDNA was synthesized using a RT^2^ First Strand Kit (Qiagen, Valencia, CA). Primers were designed as following: *Il10∶*5′-tgaattccctcggtgagaag-3′; 5′- tcactcttcacctgctccact -3′; *Il6∶*5′- ccagagatacaaagaaatgatgg-3′, 5′-actccagaagaccagaggaaat-3′; *Il4*∶5′-atggagctgcagagactgtt-3′; 5′- aaagcatggtggactagtac-3′; *Il1β*: 5′- aaatacctgtggccttgggc-3′; 5′- cttgggatccacactctccag-3′; *Nos2*∶5′-ccaagccctcacctacttcc-3′; 5′-ctctgagggctgacacaagg-3′; *Mgl1*∶5′-tgagaaaggctttaagaactggg-3′, 5′gaccacctgtagtgatgtggg-3′; *Mgl2*∶5′-ttagccaatgtgaccagctgg-3′, 5′- ggcctccaattcttgaaacct-3′; *Arg1*∶5′-ctccaagccaaagtccttagag-3′; 5′- aggagctgtcattagggacatc-3′; *Mrc2*∶5′- tacagctccacgctatggatt-3′; 5′- cactctcccagttgaggtact-3′; *Tnf*: 5′- tagccaggagggagaacaga -3′, 5′- ttttctggagggagatgtgg -3′; *Cd14∶*5′- caagtggggaacctgtcact-3′; 5′-tggcttttacccactgaacc-3′; *Ccl2∶5′-*agcaccagccaactctcact-3′; 5′- cgttaactgcatctggctga-3′; *Ccr1*∶5′- actccactccatgccaaaag-3′; 5′- ctaggacattgcccaccact-3′; *Cxcl1*∶5′- cttgaaggtgttgccctcag-3′; 5′- tggggacaccttttagcatc-3′; Realtime PCR was performed using a POWER SYBER Green PCR master mix from Applied Biosystems on ABI Prism 7500 PCR system (Applied Biosystems, Foster City, CA). The ΔCT values were used for statistical analysis for realtime-PCR experiments. The standard deviation of the fold change in gene expression for realtime-PCR data was derived by the delta method [27].

### Statistical Analysis

Differences in means were determined using one-way analysis of variance (ANOVA). The data were tested for normality using Shapiro-Wilk test prior to applying ANOVA. Multiple comparisons were conducted using the Least Significant Difference (LSD) method. All analyses were carried out using SAS® JMP software and R statistical programming language. Differences were considered significantly different when *P* was <0.05.

## Results

### IL-10 is Overexpressed in C2D-IL10 Cells

**Figure 1:**
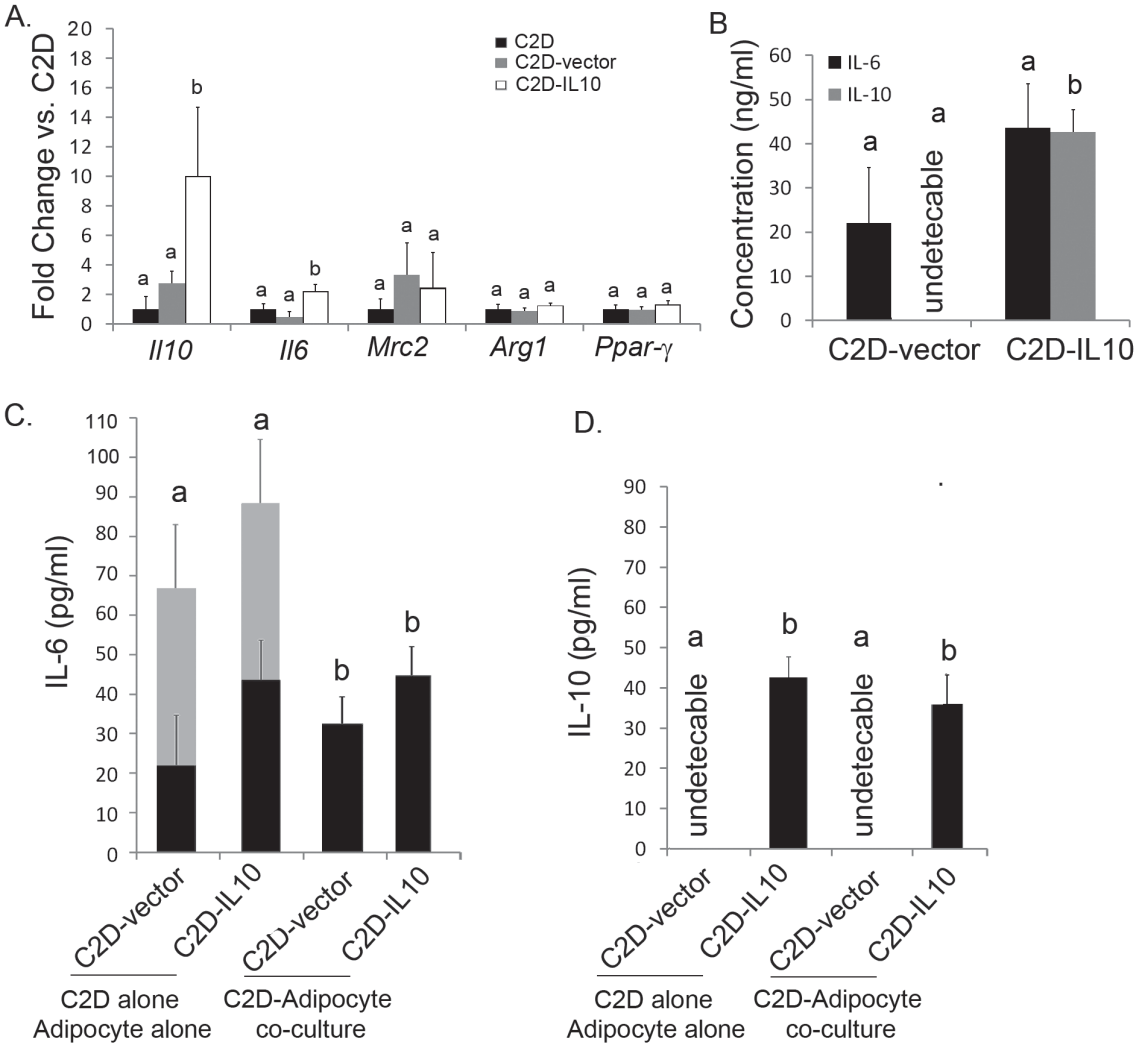
Impact of IL-10 overexpression in C2D-IL10 cells. **A)** Transcript levels were measured by realtime-PCR and compared with C2D and C2D-vector cells. **B)** Secretion of IL-6 or IL -10 in culture media was measurement by ELISA analyses. **C and D)** C2D-IL10, C2D-vector cells, or 3T3L1 adipocyte were either cultured alone (grey bar, IL-6 secretion by 3T3L1 adipocyte cultured alone; black bar, IL-6 secretion by C2D cultured alone), or 3T3L1 adipocytes were co-cultured with either C2D-IL10 or C2D-vector cells for four days as described in the Material and Methods. Overnight secretion of IL-6 or IL -10 in culture media was measured by ELISA analyses. The standard curve-based concentration lower than the sensitivity range was defined as “undetectable”. Data are presented as mean ± SEM, n = 5−6. Different characters denote *P*<0.05.

We generated a macrophage cell line that stably expresses IL-10 for use in future studies to determine whether IL-10 could play a role in regulating inflammation in adipose tissue. We previously had used C2D macrophages to assess macrophage-adipocyte interactions [4], [5], and we knew that C2D macrophages could affect the course of an infection *in vivo* after adoptive transfer [22]. Therefore, we used C2D macrophages for this purpose. We created the macrophage cell lines C2D-IL10 and C2D-vector by transfecting the C2D macrophage cell line as described in the methods. We wanted to determine the impact of the constitutive expression of IL-10 on the cells themselves and when they were in the presence of adipocytes *in vitro* or *in vivo*. Specifically, the C2D-IL10 cells and the C2D-vector cells were assessed for their expression of *Il10* transcript. We found significantly higher mRNA *Il10* levels in C2D-IL10 cells compared to parental C2D cells (Figure 1A, 10.0±4.6 fold increase; *P*<0.01). In contrast, there was not a significant increase in *Il10* transcript in C2D-vector cells compared to parental C2D cells (2.7±0.8, *P* = 0.1084). This difference was also maintained when the cells were adoptively transferred into normal mice as well. Surprisingly, there was a small biological but statistically significant increase in the proinflammatory cytokine *Il6* transcript levels in C2D-IL10 cells compared to parental C2D cells (1.0±0.4 vs. 2.2±0.4, *P* = 0.0410), but *Il6* mRNA levels were not elevated in C2D-vector cells compared to parental C2D cells (1.0±0.4 *vs*. 0.5±0.4, *P* = 0.3370). Transcriptional expressions of other known macrophage-related genes *Mrc2*, *Arg1* and *Ppar-γ* were not different among C2D-vector cells and C2D-IL10 cells compared to C2D.

We performed ELISA to determine if IL-10 secretion was consistent with *Il10* transcript levels in C2D-IL10 and C2D-vector macrophage cells. Secreted IL-1β and TNF-α were undetectable in both C2D-IL10 and C2D-vector cells. In contrast, we observed significant IL-10 secretion by C2D-IL10 cells compared to C2D-vector cells (Figure 1B), while the amounts of IL-6 in overnight culture secretions of C2D-IL10 cells was not different than that of the C2D-vector cells (Figure 1B). These data also suggest that the increased amount of *Il-6* transcript in C2D-IL10 (Figure 1A) did not translate to IL-6 secretion.

We previously found that C2D macrophages would secrete cytokines in response to co-culture with 3T3-L1 adipocytes [5]. Therefore, we wanted to determine if overexpression of *Il10* in C2D-IL10 cells affected the macrophages in these macrophage-adipocyte interactions. We assessed secretion of IL-10 and IL-6 in 3T3-L1 adipocyte-C2D-IL10 co-cultures or adipocyte-C2D-vector co-cultures. The amounts of IL-6 and IL-10 were compared to the sum of the secretion of equal number of adipocytes (Figure 1C, gray bar) and macrophages (Figure 1C, black bar) cultured alone. IL-6 concentrations were significantly reduced in co-cultures of C2D-vector and C2D-IL10 cells plus adipocytes compared to the sum of independent cultures of adipocytes and C2D-IL10, or C2D-vector cells, which may be due to the increased consumption of IL-6 in co-cultures as we discussed before [5]. The IL-10 concentration was undetectable in C2D-vector-adipocyte co-cultures, while it was 44.7±7.2 pg/ml in C2D-IL10-adipocyte co-culture (Figure 1D). IL-1β and TNF-α levels were undetectable in both C2D-vector-adipocyte and C2D-IL10-adipocyte co-cultures (not shown).

### Elevation of C2D-IL10 M2 Surface Markers and M1 and M2 Cytokine Gene Transcripts in Response to 3T3L1 Adipocytes

**Figure 2:**
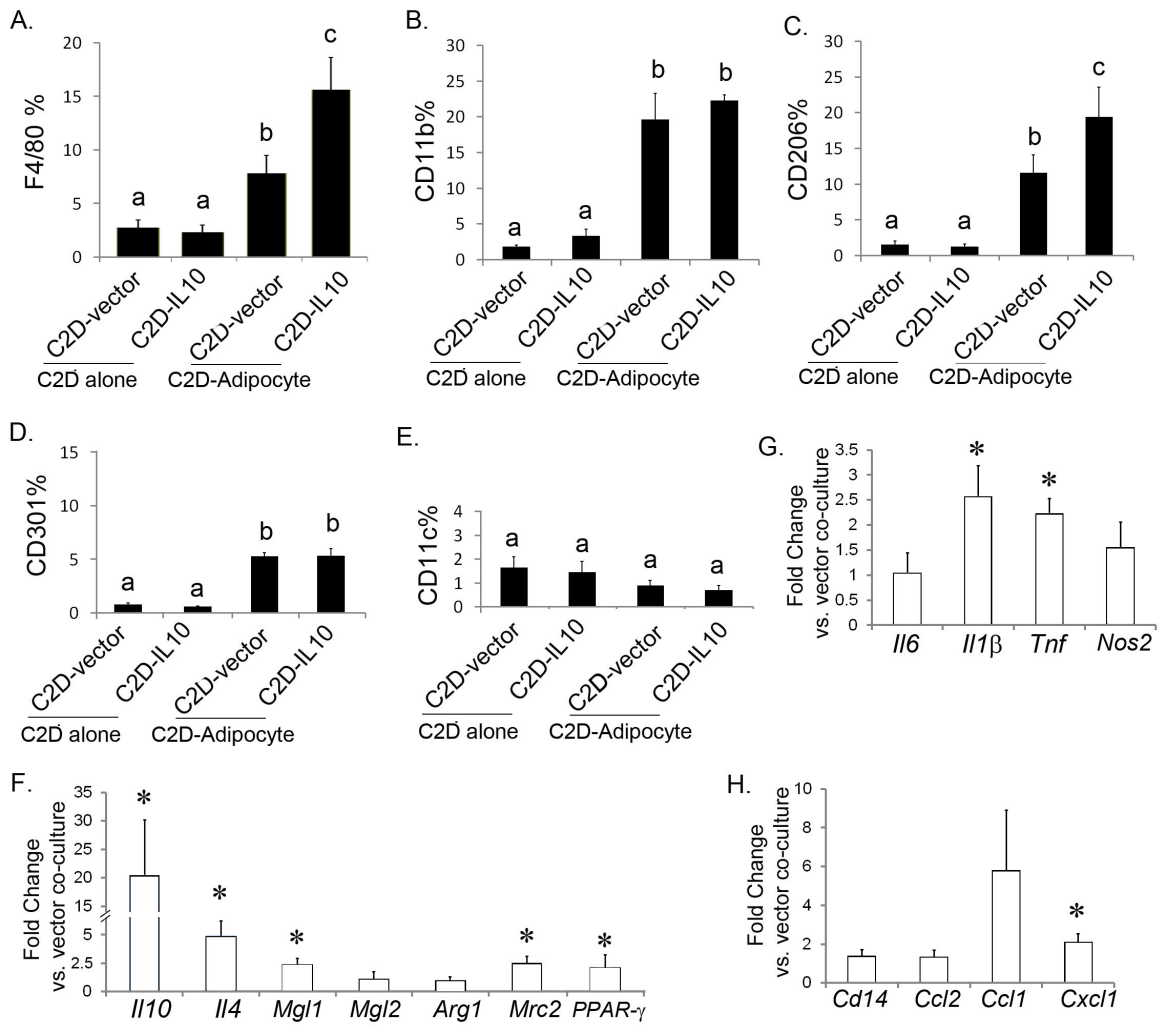
C2D-IL10 cells elevated the expression of M2 marker proteins in response to 3T3L1 adipocytes. C2D-IL10 or C2D-vector cells stained with CFDA SE were either cultured alone, or co-cultured with differentiated 3T3L1 adipocytes for four days as described in the Material and Methods. **A–E)** Macrophage surface markers were analyzed on the CFDA SE+ population by flow cytometry. Different characters denote *P*<0.05. **F and G)** Transcripts differences between C2D-IL10-adipocyte and C2D-vector-adipocyte were measured by real-time PCR. * P<0.05. Data are presented as mean ± SEM, n = 5−6.

Because we are interested in macrophage-adipocyte interactions [4], [5], we investigated how IL-10 overexpression impacted macrophage phenotypes in C2D-IL10-adipocyte co-culture, compared to C2D-vector-adipocyte co-culture. In order to visualize and identify the C2D-IL10 or C2D-vector cells, the cells were labeled with CFDA-SE prior to the 4 day co-culture period when the cell number of C2D-IL10, or C2D-vector, (CFDA-SE^+^) and 3T3L1 adipocytes(CFDA-SE^−^) were cultured together in equal numbers. F4/80 and CD11b expression in C2D-vector and C2D-IL10 cells cultured alone was very low. However, C2D-vector cells and C2D-IL10 cells co-cultured with 3T3-L1 adipocytes expressed higher levels of F4/80 (Figure 2A) and CD11b (Figure 2B). Moreover, F4/80 expression was higher on C2D-IL10 cells co-cultured with 3T3-L1 adipocytes compared to co-cultured C2D-vector cells (Figure 2A).

Previous studies have identified the classical myeloid dendritic cell markers CD11c, C-type lectin receptors (CLR) CD206 and CD301 as surface markers on adipose tissue M1 or M2 macrophages [8], [10], [12], [28], [29], [30]. Therefore, we analyzed our cells for the expression of CD11c, CD206 and CD301 after co-culture with 3T3-L1 cells. Co-culture with adipocytes induced expression of CD206 (Figure 2C) and CD301 (Figure 2D), but not CD11c (Figure 2E), on both C2D-vector and C2D-IL10 cells. Expression of CD206 was significantly higher in co-cultured C2D-IL10 cells than in co-cultured C2D-vector cells (Figure 2C, 11.6% ±2.5% vs. 19.4±4.1%, *P* = 0.005).

Because co-culture of C2D-IL10 with 3T3-L1 adipocytes caused changes in some of the surface phenotype antigens (*e.g.* F4/80 and CD206), we hypothesized that overexpression of IL-10 in C2D macrophages might change the inflammatory status of the co-culture with adipocytes. Indeed, transcript levels of *Il10, Il4, Mgl1* and *Mrc2* were significantly enhanced in C2D-IL10-adipocyte co-cultures versus C2D-vector-adipocyte co-cultures (Figure 2F); consistent with the fact that C2D-IL10-adiocyte co-culture expressed more CD206 compared to C2D-vector-adiocyte co-culture. Transcript levels of pro-inflammatory cytokines, *Tnf, Il1β* (Figure 2G) and *Cxcl1* (Figure 2H), of C2D-IL10-adipocyte co-cultures were also slightly higher than those of C2D-vector-adipocyte co-cultures.

### Impact of C2D-IL10 Overexpression on Macrophage Phenotype in Epididymal Adipose Tissue

**Figure 3:**
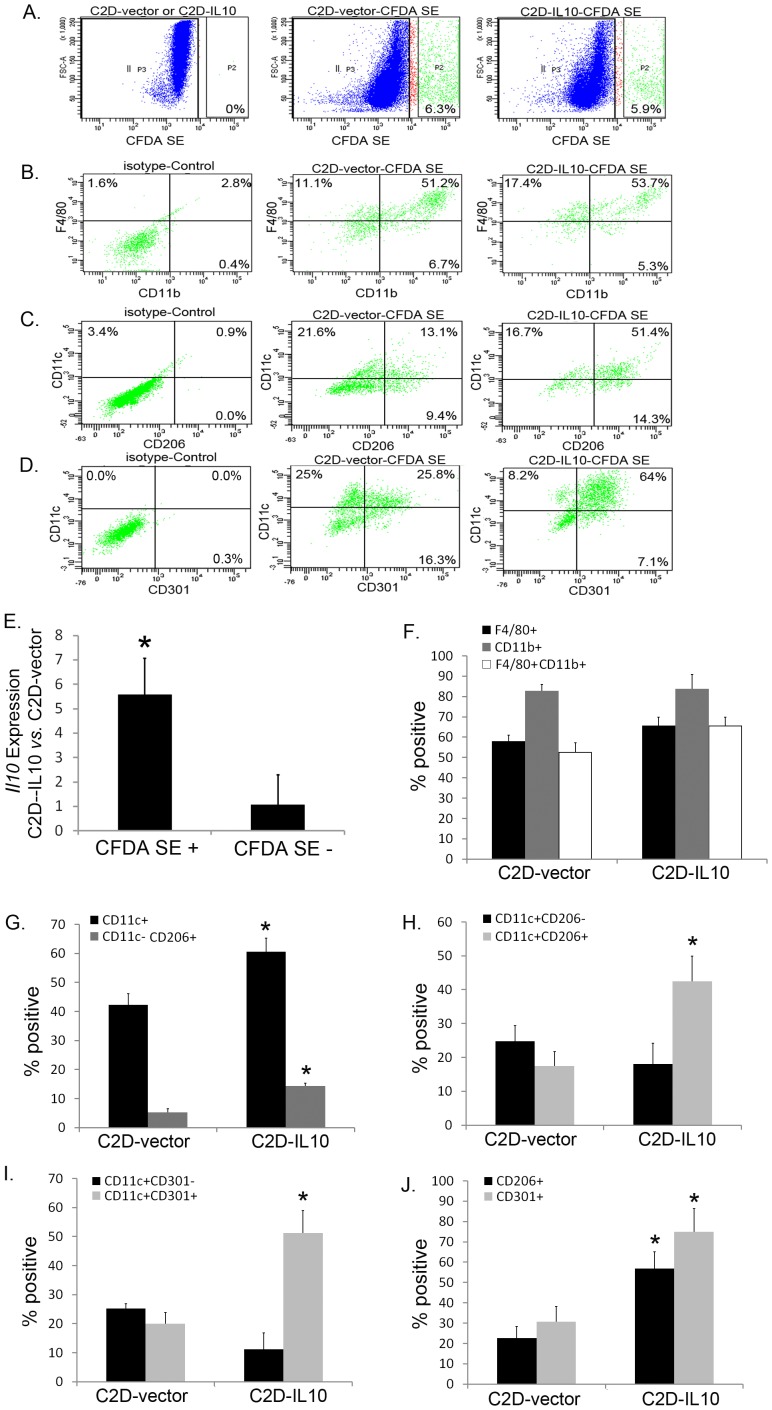
Isolated C2D-IL10 cells from adipose tissue expressed a higher percentage of CD11c, CD206 and CD301, compared to isolated C2D-vector cells. CFDA SE labeled C2D-IL10 or C2D-vector cells were adoptively transferred to lean mice by peritoneal injection. Seventy-two hours later, the epididymal adipose tissue SVF was collected and the newly-infiltrated C2D-IL10 or C2D-vector cells were identified by CFDA SE positive. (A–D) Fresh isolated SVC containing injected C2D-IL10 or C2D-vector cells were immunostained with the specific antibody (anti-F4/80-PE-Cy7, anti-CD11b-Alexa Fluor® 647, anti-CD11b-PE, anti-CD206- Alexa Fluor® 647 and anti- anti-CD301-Alexa Fluor® 647) or corresponding isotype control. Labeled cell surface proteins were assessed by BD LSR II flow cytometer. Shown images are example scatter plots. (E) Newly-infiltrated C2D-IL10 or C2D-vector cells were identified by CFDA SE positive and isolated by cell sorting. Expression of *Il10* in either CFDA SE^+^ or CFDA SE^−^ was assessed by realtime-PCR. (F–J) The macrophage surface makers of CFDA SE^+^ cells were assessed by flow cytometry (Example Flow Cytometry scatter plots were shown in panel A–D). Data are presented as mean ± SEM, n = 3−5. * *P*<0.05.

Because there were cell surface and transcriptional changes in the C2D-IL10 macrophage phenotype compared to C2D-vector cells during 3T3L1 co-culture, we wanted to determine if a similar impact would be seen *in vivo.* We previously isolated C2D macrophages from the SVF of epididymal adipose tissue 72 hours after adoptive transfer and found that C2D macrophage cells expressed Ly6C, CD11b and F4/80 [4], [5]. We found that the isolated SVF consisted of around 90%–94% CFDA SE negative cells and 6–10% of CFDA SE positive cells after adoptive transfer of both cell types. Therefore, we isolated the SVF C2D-vector cells or the SVF C2D-IL10 cells by identifying the CFDA SE positive via cell sorting. The expression of *Il10* was assessed by realtime-PCR. *Il10* transcription level was significantly higher in isolated SVF C2D-IL10 cells than in SVF C2D-vector cells (5.58±1.48 fold change, *P* = 0.030, Figure 3E), confirming that the overexpression of IL-10 in SVF C2D-IL10 cells was maintained *in vivo.* The *Il10* expression level in SVF CFDA SE negative population was not different between mice injected with C2D-IL10 cells and mice injected with C2D-vector cells (1.06±1.24 fold change, *P* = 0.951, Figure 3E).

Both C2D-vector cells and the C2D-IL10 cells, isolated from visceral white adipose tissue, expressed very high levels of CD11b and F4/80 (Figure 3B and F). There was a consistent percentage of CD11b^+^F4/80^+^ after adoptive transfer of both cell types (52.6% ±4. 6% vs. 65.6±4.2%, *P* = 0.0738).

Unlike when C2D-vector and C2D-IL10 cells were co-cultured with adipocytes *in vitro*, both cell types had higher levels of CD11c, with C2D-IL10 cells expressing more (Figure 3C and G, 42.3% ±3.8% vs. 60.6±4.6%, *P* = 0.02). We also observed that 13.2% of C2D-IL10 cells were CD11c^−^CD206^+^, compared to only 6.5% of C2D-vector cells are (Figure 3C and G, 6.5±2.0% vs. 13.2±2.04%, *P* = 0.0008). To further analyze the subtypes of CD11c^+^ cells, we observed that 42.3% of C2D-IL10 macrophage were CD11c^+^CD206^+^ (M1b phenotype); however, only 17.7% of C2D-vector cells expressed CD11c^+^CD206^+^ (Figure 3C and H, *P* = 0.032). The percentage of CD11c^+^CD206^−^ (M1a phenotype) in isolated C2D-IL10 cells was not different from isolated C2D-vector cells (Figure 3C and H, 24. ±4.5% vs. 18.1±6.1%, *P* = 0.41). The percentage of CD206^+^ cells among total isolated C2D-IL10 cells was also much higher than those among isolated C2D-vector cells (Figure 3C and J, 22.7±5.–5% vs. 56.9±7.9%, *P* = 0.0008).

We also examined the expression of CD301 within the CD11c^+^ population of C2D-IL10 and C2D-vector cells isolated from the SVF after adoptive transfer. The percentage of CD11c^+^CD301^+^ C2D-IL10 cells was more than two fold higher than C2D-vector cells (Figure 3D and I, 20±3.8% vs. 51.2±7.6%, *P* = 0.0176). There were no differences between transferred CD11c^+^CD301^−^ in C2D-IL10 and C2D-vector cells (Figure 3D and I, 25.2±1.7% vs. 11.1±5.8, *P* = 0.0879). Similar to that of CD206, the percent expression of CD301 was much higher among transferred C2D-IL10 cells than C2D-vector cells (Figure 3D and J, 30.6±6.3% vs. 74.9±9.3%, *P* = 0.0428). These results suggest that IL-10 overexpression led to a phenotypic change in the C2D-IL10 cells when they trafficked to the adipose tissue environment.

### Phenotypic Changes in Adipose Tissue Isolated C2D-IL10, Compared to C2D-vector Macrophage Cells, is not Due to Altered Adipose Tissue Inflammation

**Figure 4:**
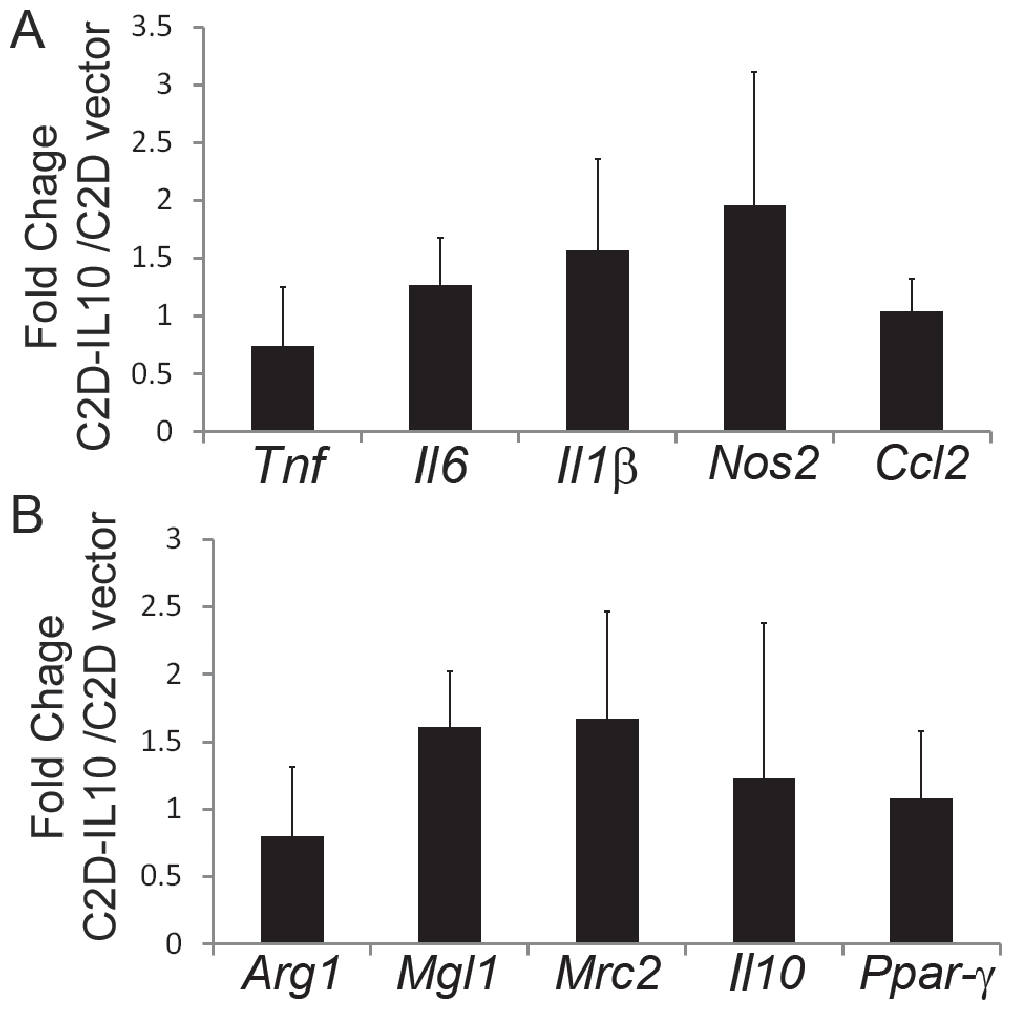
Effect of C2D-IL10 cells on expression of inflammation-related genes in epididymal fat tissue. CFDA SE labeled C2D-IL10 or C2D-vector cells were adoptively transferred to lean mice by intraperitoneal injection. Seventy-two hours later, the epididymal adipose tissue was collected and the total RNA was extracted. (A) Transcript levels of *Tnf, Il6, Il1β, Nos2* and *Ccl2* were analyzed by qRT-PCR. Data are presented as mean ± SEM, n = 4−5. (B) Transcript levels of *Arg1, Ngl1, Mrc2, Il10* and *Ppar-γ* were analyzed by qRT-PCR. Data are presented as mean ± SEM, n = 4−5.

It is possible that adipose tissue trafficking C2D-IL10 or C2D-vector cells could cause different levels of tissue inflammation, which, in a feedback loop, lead to a macrophage phenotypic switch. To determine the impact of newly infiltrated C2D-IL10 or C2D-vector on adipose tissue inflammation, we examined the adipose tissue inflammatory status by measuring adipose tissue pro-inflammatory gene transcripts including *Il6*, *Il1β, Tnf, Nos2* and *Ccl2,* and M2 marker genes including *Il10*, *Arg1, Mrc2, Mgl1* and *Ppar-γ.* None of the transcripts measured were significantly different between C2D-IL10 recipient mice and C2D-vector recipient mice (Figures 4A and B).

### Recombinant IL-10 Treatment on C2D Cells Co-cultured with 3T3L1 Adipocytes did not Increase Expression of CD11c, CD206 and CD301

**Figure 5:**
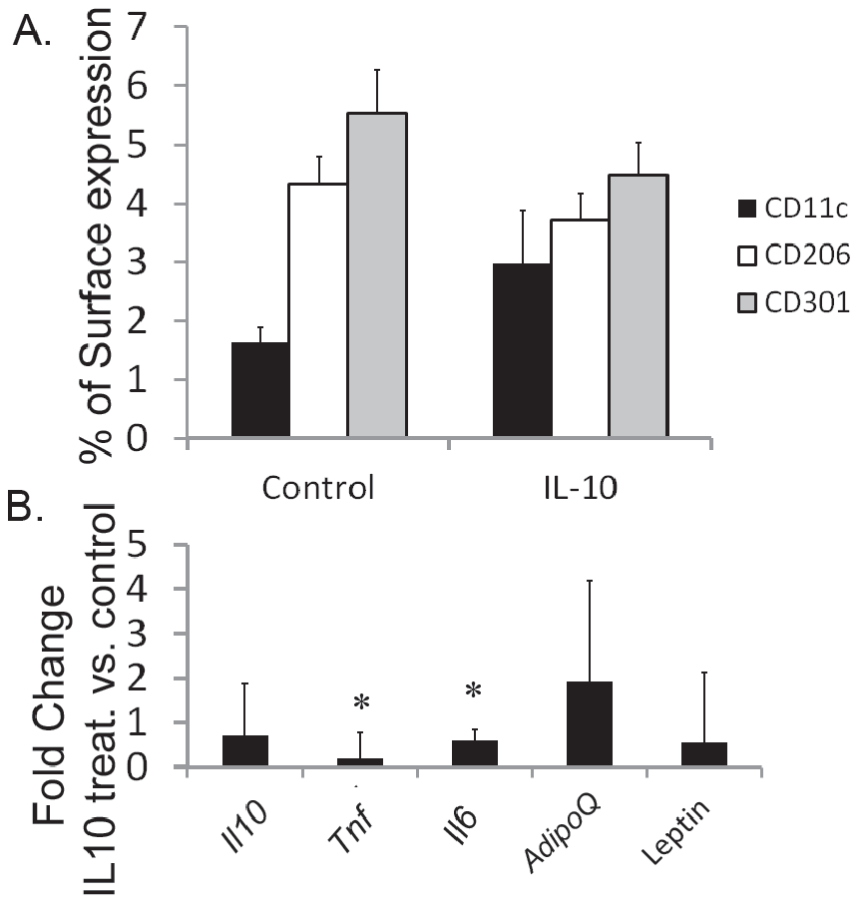
Recombinant IL-10 treatment on C2D cells co-cultured with 3T3L1 adipocytes decreased expression of *IL-6* and *Tnf*. Co-cultured C2D-adipocytes were treated with or without IL-10 for six hours. Co-cultured C2D-adipocytes were suspended in PBS: half of the cells were analyzed by flow cytometry for surface markers (A) and the other half of the cells were analyzed by real-time PCR (B). Data are presented as mean ± SEM, n = 4−5. *P<0.05.

Because the C2D-IL10 cells in an adipose tissue environment had a different phenotype compared to C2D-vector cells, we determined if the effect was due to the levels of IL-10 secreted by the C2D-IL10 cells. We tested if recombinant mouse IL-10 treatment of C2D (labeled with CFDA SE) co-cultured with 3T3L1 cells would cause any phenotypic changes of C2D cells. In particular, we treated the cells with a relatively high starting concentration of IL-10 (1000pg/ml) to provide a drastic comparison to the secretory concentration of IL10 in C2D-IL10-adipocyte co-cultures (about 40 pg/ml). It is known that the half-life of mouse IL-10 is 20 minutes [31]. Therefore, IL-10 was replenished every two hours for three times to ensure that the concentration of IL10 was greater than 40 pg/ml for at least 6 hours. Regardless, IL-10 treatment did not increase surface expression of CD11c in CFDA SE-positive C2D cells co-cultured with 3T3L1 adipocytes (Figure 5A). Similarly, surface expression of CD206 and CD301 on C2D cells co-cultured with 3T3L1 adipocytes was not changed by the 6-hour IL-10 treatment (Figure 5A). We were concerned that the 6-hour treatment window was not long enough to induce a change in surface protein expression, thus, we also tested if the high-dose IL-10 treatment during C2D-adipocyte co-culture would affect the mRNA levels of the inflammatory cytokines *Il10, Il6, Il1β* and *Tnf* and the adipokines *AdipoQ* and *Lep*. There was a lower mRNA expression of *Il6* and *Tnf* in C2D-adipocyte co-cultures treated with IL-10 than the untreated controls (Figure 5B). *Il10, Il1β, AdipoQ* and *Lep* transcript levels were unchanged in those same co-cultures (Figure 5B). Therefore, there was only a minor impact of high-dose IL-10 treatment on C2D macrophages during a six-hour adipocyte co-culture.

## Discussion

We successfully generated a macrophage cell line, C2D-IL10 that constitutively secretes IL-10 at significant levels. This novel tool allowed us to examine the impact of the constitutive expression of IL-10 on the C2D-IL10 macrophages either by themselves or when they were co-cultured with adipocytes and adipose tissue. One of the concerns when overexpressing a cytokine in a cell is the autocrine impact of that cytokine. This does not appear to be a significant issue because there were similar levels of F4/80, CD11b, CD206, CD301 and CD11c on C2D-IL10 compared to C2D-vector cells. There were also similar transcript levels of *Mrc2*, *Arg1* and *Ppar-γ* and similar secretion of IL-6. It was clear that the C2D-IL10 cells were making additional IL-10 cytokine as evidenced by increased transcript and IL-10 secretion (Figure 1), but the lack of change in CD206 and CD301 molecules associated with M2 macrophages (Figure 1A ) suggests that the additional IL-10 was not driving the C2D-IL10 cells to be more “M2-like” when the cells were in culture alone.

One purpose of creating C2D-IL10 was to determine what impact constitutive expression of IL-10 would have on the macrophages when they came in contact with adipocytes or an adipose tissue environment. IL-10 has been reported to promote the differentiation of M2 macrophages during microbial infection [32], [33]. We observed increased expression of F4/80 and CD206 on C2D-IL10 macrophages and higher macrophage specific *Mgl1* and *Mrc2* level of the co-cultured C2D-IL10-3T3L1 adipocytes cells compared to the co-cultured C2D-vector-3T3L1 adipocytes cells (Figure 2). The slightly higher expression of *Il-1β, Tnf* and *Cxcl1* (about 1 fold change) in the C2D-IL10-adipocyte co-culture compared to C2D-vector-adipocyte co-culture (Figure 2) is also consistent with observations that M2 macrophages express a low level of inflammatory (M1) cytokines [34], [35], [36], [37]. Consistently, we found twice as many CD11c^−^CD206^+^ (marker of M2) C2D-IL10 cells compared to C2D-vector cells after isolation from adipose tissue (Figure 3G, gray bars). Therefore, our data indicate that constitutively overexpression of IL-10 promotes the M2 phenotype in C2D-IL10 macrophages in adipose tissue.

Even though more C2D-IL10 cells, comparing to C2D-vector cells, were CD11c^−^CD206^+^ in the presence of 3T3L1 adipocytes, the IL-10 production in C2D-IL10 cells also seemed to promote a macrophage bi-directional phenotype switch as well. They not only increased cell surface CD206 and CD301 expression but also increased CD11c expression when they migrated into a more complicated *in vivo* adipose tissue environment. It is also interesting that the higher percentage of CD11c^+^ cells in the C2D-IL10 population versus the C2D-vector population (Figure 3G, black bar,18.3% more) was totally due to the abundance of CD11c^+^CD206^+^ cells (Figure 3H, grey bar; 24.6% more). It was reported that CD11c^+^CD206^+^ ATMs, classified as M1b, have features of both classically activated macrophages and of alternatively activated macrophages, as evidenced by the high levels of *Il10* mRNA and IL-10 secretion [38]. It is unknown what mechanism triggers the differentiation of CD11c^+^ macrophages into CD206^+^ and CD206^−^ subpopulations. Our data suggest that IL-10 is involved in stimulating the CD11c^+^ macrophage to express more CD206, or an intermittent stage between M2 and M1.

Clearly, IL-10 alone is not enough to complete the phenotypic switch of either M1 or M2 macrophages; additional signals are required. It is possible that injected C2D cells, which migrated into normal adipose tissue, lacked stimulation from the lipotoxic environment of obese mice that triggers the signaling cascade of proinflammatory cytokines [39], [40]. It is also possible that the number of adipose tissue recruited C2D-IL10 macrophages (only about 6–10% of SVCs) was not large enough to affect the local inflammation. Therefore, C2D-IL10 macrophage cells maintained a mixed M1/M2 phenotype because of the missing “signal”.

Our study provides direct evidence to suggest that IL-10 may regulate the macrophage phenotype *in vivo*. In other studies, overexpression of IL-10 systematically increased adipose tissue M2 macrophages, as evidenced by enhanced expression of *Mrc2, Ym1, CD163*, and *CD209a* in mouse epididymal fat [10]. These data suggest a role for IL-10 on ATMs heterogeneity via either enhanced M2 macrophage recruitment to the tissue or direct regulation of the macrophage phenotypic switch. Our data for the first time provide evidence to show that IL-10 directly impacts ATM phenotype. In another study, adoptive transfer of IL-10-knockout bone marrow cells (*IL-10-*KO BMT) to lethally irradiated wild-type recipient mice did not decrease the mRNA level of *Mgl1* in bone marrow-derived macrophages 12 weeks after transfer [10]. These data did not exclude a role of IL-10 in regulating macrophage phenotype, because the plasma IL-10 level was not different in the *IL-10-*KO BMT mice compared to the wildtype BMT mice [41]. We, by directly tracking the physiological changes of C2D *in vivo*, confirmed that overexpression of IL10 could increase surface CD11c, CD206 and CD301 on adipose tissue isolated C2D-IL10 cells compared to isolated C2D-vector cells. We excluded the possibility that the phenotypic changes seen in C2D-IL10 cells isolated from adipose tissue were caused by enhanced adipose tissue inflammation induced by C2D-IL10 cells because adipose tissue had unchanged adipose tissue pro-inflammatory gene transcript levels in C2D-IL10 recipient mice compared to C2D-vector recipient mice.

Although some suggest that ELISA is inappropriate for quantification of blood IL-10 concentrations, the mean mouse serum concentration of IL-10 is 32.4 pg/ml [31], which is close to what is secreted by our C2D-IL10 cells or C2D-IL10-in adipocyte co-culture. Therefore, we suggest that the C2D-IL10-adipocyte co-culture model may be a more physiological environment to study the role of IL-10 on macrophage-adipocyte interactions.

High dose recombinant IL-10 treatment of C2D-3T3L1 adipocytes caused decreased expression of *Il6* and *Tnf (* Figure 5B), which is consistent with a previous report [8]. However this contrasts our observations from both *in vitro* co-culture experiments or our *in vivo* observations. We also realize that acute and high dosage treatment of *in vitro* cultures is physiologically different than constant secretion of cytokine over long periods of time. However, these data help assuage concerns that higher concentrations of IL-10 would have a different impact on C2D macrophages compared to a lower dose. Nevertheless, this also raises one concern about the narrow or opposite impact of C2D-IL10 macrophages on inflammatory cytokines in adipose tissue or in co-culture with 3T3L1 adipocytes. It is possible that the secreted concentration of IL-10 by C2D-IL10 was too low to cause similar physiological changes *in vivo* as *in vitro* (when the number of adipocytes was equal to that of C2D-IL10). Actually, Fujisaka *et al*. also did not observe different levels of adipose tissue *Il6* and *Tnf* in either lean or obese mice injected with Ad-hIL-10 (IL-10) when compared with those injected with a control vector [10]. It is also possible that *in vivo*, IL-10 over-secretion by C2D-IL10 induces secretion of other unknown factors by an intermediate (other immune cells or adipocytes) that might trigger or promote the phenotypic switch of macrophage, while *in vitro* co-culture experiment lacks such possibility. Additional experiments will be needed to test this hypothesis.

In conclusion, our findings suggest that constitutive expression of IL-10 in C2D-IL10 macrophages affects the C2D-IL10 macrophage phenotypic switch in adipose tissue environments. Our study raises additional questions about whether C2D-IL10 cells will have an impact on the obese adipose tissue itself, a host’s inflammatory status during processes such as diet-induced obesity or in mice that are genetically predisposed toward obesity. We now have important tools to investigate these questions.
